# The Hidden Dangers of Plant-Based Diets Affecting Bone Health: A Cross-Sectional Study with U.S. National Health and Nutrition Examination Survey (NHANES) Data from 2005–2018

**DOI:** 10.3390/nu15071794

**Published:** 2023-04-06

**Authors:** Yi Zheng, Jiacheng Wang, Yawen Wang, Kelin Xu, Xingdong Chen

**Affiliations:** 1 State Key Laboratory of Genetic Engineering, Human Phenome Institute, Zhangjiang Fudan International Innovation Center, National Clinical Research Center for Aging and Medicine, Huashan Hospital, Fudan University, Shanghai 200032, China; 2 Department of Epidemiology, School of Public Health, The Key Laboratory of Public Health Safety of Ministry of Education, Fudan University, Shanghai 200032, China; 3 Department of Biostatistics, School of Public Health, The Key Laboratory of Public Health Safety of Ministry of Education, Fudan University, Shanghai 200032, China; 4 Fudan University Taizhou Institute of Health Sciences, Taizhou 225300, China; 5 Yiwu Research Institute of Fudan University, Yiwu 322000, China

**Keywords:** bone mineral density, plant-based diet index, osteopenia, osteoporosis, NHANES, dietary pattern

## Abstract

The plant-based dietary pattern has been recommended for its potential health and environmental benefits, but its association with bone loss needs to be further explored. This study aimed to investigate the association between three plant-based diet indexes and bone loss in 16,085 adults, using data from the National Health and Nutrition Examination Survey. Three plant-based diet indexes (PDI, hPDI, and uPDI) were calculated from two NHANES 24-h dietary recall interviews, to characterize a plant-based diet. A multinomial logistic regression model was used to estimate the odds ratios (OR) and 95% confidence intervals (95% CI). Higher hPDI and PDI were associated with increased risk of bone loss (OR_Q5 vs. Q1_ = 1.50; 95% CI: 1.24–1.81 for hPDI; OR_Q5 vs. Q1_ = 1.22; 95% CI: 1.03–1.45 for PDI), while higher uPDI was associated with increased risk of osteoporosis (OR_Q5 vs. Q1_ = 1.48; 95% CI: 1.04–2.11). A harmful association between plant-based diet indexes (hPDI and PDI) and osteopenia was observed at the lumbar spine rather than the femoral neck. We conducted several sensitivity analyses to ensure the robustness of results, including subgroup analysis, exclusion of people taking anti-osteoporotic and estrogenic drugs, further adjustment for menopausal status, corticosteroid usage, and dietary supplements, and calculation of E-value. Our study demonstrates the deleterious effects of a plant-based diet on bone health and emphasizes the importance of a balanced diet.

## 1. Introduction

Osteoporosis is a commonly occurring metabolic bone disease characterized by a reduction in bone mineral density (BMD) and the deterioration of bone microarchitecture, frequently leading to increased risk of bone pain and fragility fractures [1,2]. This disease affects over 200 million individuals globally, and its incidence continues to rise annually, particularly among middle-aged and elderly populations [3,4]. The annual medical cost of osteoporosis-related fractures in the United States alone is estimated to be around $17.9 billion annually, imposing a heavy economic burden [5]. Consequently, preventing osteoporosis has become a significant public health concern. In addition, although medication is an effective treatment for osteoporosis, the percentage of patients receiving medication treatment remains low due to the fragmented nature of the healthcare system and concerns about medication side effects [6,7]. Lifestyle changes, such as increasing physical activity and modifying dietary habits, may provide a more feasible and effective approach to improving bone health [8].

Plant-based dietary patterns, characterized by higher intake of plant foods and lower consumption of animal foods, have been widely recommended as healthy dietary options [9,10,11,12]. A plant-based diet has been shown to improve the diversity and composition of the intestinal microbiota, leading to increased production of specific metabolites that exert beneficial effects on host health, including at the intestinal and systemic levels [13]. Accumulating evidence suggests that a plant-based diet plays a positive role in preventing chronic diseases such as type 2 diabetes mellitus (T2DM), hypertension, and cardiovascular disease [10,14,15]. However, a plant-based diet has been found to include lower levels of calcium, vitamin D, vitamin B-12, protein, and n-3 fatty acids, which are all crucial for maintaining bone health [16]. As a result, individuals following plant-based diets may exhibit lower BMD and higher risk of fractures. In a recent study of participants in the 2007–2010 National Health and Nutrition Examination Survey (NHANES), self-identified vegetarians had significantly lower BMD than non-vegetarians [17]. Additionally, a meta-analysis found that individuals following a plant-based diet exhibited lower BMD and higher rates of fractures in the femoral neck and lumbar spine than those following an omnivorous diet [18]. Consequently, caution should be exercised when transitioning from an animal-based diet to a plant-based diet, particularly for individuals shifting towards a vegan diet.

In previous studies, plant-based diets were typically defined as “vegetarian” diets, with participants being divided into two groups: those who consume any animal-based products and those who do not [17,19,20]. However, transitioning to a completely animal-free diet presents a considerable challenge for many individuals, attributable to cultural acculturation and the affordability of plant-based foods [21,22,23]. The pressure to assimilate into American society and adapt to the fast-paced lifestyle can make it challenging to avoid consuming animal-based foods, particularly during social gatherings [21,22]. Furthermore, the cost of plant-based products can be higher than that of animal products in certain regions, exerting a notable influence on people’s dietary choices [23]. Thus, from a public health perspective it is essential to investigate the impact on osteoporosis caused by gradually increasing the consumption of plant-based foods while simultaneously reducing the intake of animal-based products.

Recently, Satija et al. conceptualized a graded dietary pattern comprising three plant-based dietary indexes: overall plant-based diet index (PDI), healthy plant-based diet index (hPDI), and unhealthy plant-based diet index (uPDI) [10]. The PDI reflects an overall trend towards a progressive increase in the proportion of plant-based foods, accompanied by a gradual decrease in the intake of animal-based foods. The hPDI and uPDI distinguish between healthy and unhealthy plant-based foods, addressing the limitations of previous studies that treated all plant-based foods equally [24]. These three plant-based indexes provide a more comprehensive approach to assess nutrient density and the impact of dietary changes on health, guiding individuals toward more sustainable and healthier diets [25]. However, few studies have used the three indexes to examine the association between a plant-based diet and BMD. Despite some initial research conducted in Iranian and Chinese populations, the association between plant-based dietary indexes and BMD is still not well understood, and the findings are inconsistent [26,27]. Further studies with larger sample sizes and diverse populations are needed to better elucidate this relationship and its potential implications.

Given the theoretical background outlined above, the aim of this study was to investigate the potential relationship between plant-based dietary indexes and osteopenia/osteoporosis in the adult population of the United States. Ultimately, the findings of this study may provide a valuable theoretical foundation for the development of strategies to prevent osteoporosis.

## 2. Materials and Methods

### 2.1. Study Population

**Figure 1:**
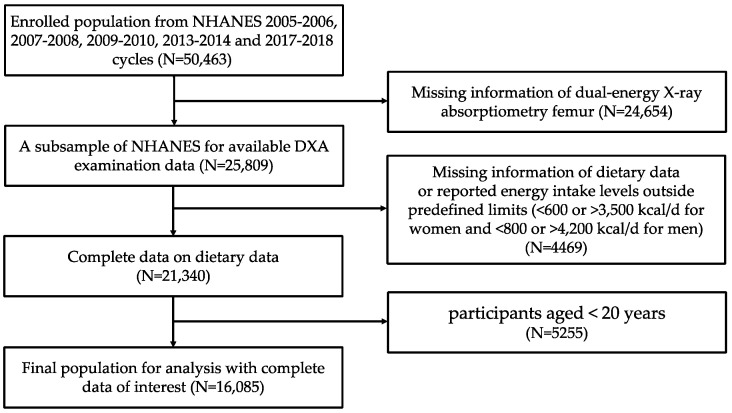
Flow diagram of inclusion and exclusion criteria.

The present study utilized publicly available data from the NHANES, affiliated with the National Center for Health Statistics (NCHS) of the Centers for Disease Control and Prevention (CDC). The NHANES was designed to evaluate nutrition status and the prevalence of disease in the US population. Due to the unavailability of BMD data for the femoral neck in 2011–2012 and 2015–2016, subject information was collected from five 2-year NHANES cycles (2005–2006, 2007–2008, 2009–2010, 2013–2014, and 2017–2018). Inclusion criteria were as follows: (i) participants aged ≥20 years; (ii) participants with complete BMD and dietary interview data; (iii) participants with reported energy intake levels within predefined limits (≥600 and ≤3500 kcal/d for women and ≥800 and ≤4200 kcal/d for men). Participants with energy intakes outside these limits were excluded from the analysis, as such extremes in energy intake may not represent the general population and could introduce bias into the results. We identified 50,463 potential participants from the five NHANES cycles (NHANES 2005–2006, 2007–2008, 2009–2010, 2013–2014, and 2017–2018). After excluding the participants who did not meet the inclusion criteria, we ultimately recruited 16,085 participants. The detailed depiction of the inclusion and exclusion process is illustrated in Figure 1. All study participants provided informed consent, and the Ethics Review Board of the NCHS approved all study procedures.

### 2.2. Bone Mineral Density Assessment

All subjects had BMD (g/cm^2^) measured at the lumbar spine (L1–L4) and the femoral neck using a dual-energy X-ray absorptiometry densitometer (Hologic QDR-4500A; Bedford, MA, USA). Participants were excluded from the DXA examination if they satisfied any of the following criteria: (i) participants who were pregnant; (ii) participants who weighed more than 450 pounds; (iii) participants who had a self-reported history of radiographic contrast material in the past 7 days; (iv) participants with bilateral hip fractures, replacements, or pinning. As recommended by the World Health Organization, the mean BMD of non-Hispanic white females aged 20–29 years from NHANES III was used as the reference group for the femoral neck, while the mean BMD of non-Hispanic white females aged 30–39 years from NHANES was used as the reference group for the lumbar spine [28,29]. In addition, the 16,085 participants were classified into three categories (normal, osteopenia, and osteoporosis) based on the minimum BMD T-score of the two measuring sites. Osteopenia was diagnosed according to a BMD T-score between −1.0 and −2.5, while osteoporosis was diagnosed according to a BMD T-score ≤ −2.5.

### 2.3. Plant-Based Diet Index

Dietary intake data were collected from two NHANES 24-h recall interviews and extracted after conversion to the respective food equivalents in the food-pattern-equivalence database. Additionally, dietary intake was estimated using the average of two 24-h recall data. To calculate the three plant-based diet indexes, we assessed the intake of 15 food groups, which were divided into three categories: healthy plant-based foods, unhealthy plant-based foods, and animal-based foods. We assigned positive or reverse scores to each food item based on the quintile of intake. Details of the 15 food groups and scoring rules are shown in Supplementary Table S1. Each subject’s scores were summed to obtain a score for each index, with a theoretical range of 15 to 75. Finally, these index variables were treated as continuous (per 10-unit increment) and categorical (in quintiles), respectively.

### 2.4. Covariates

Participants’ demographical characteristics (age, sex, ethnicity, educational level, poverty income ratio (PIR), body mass index (BMI), and marital status), lifestyle (smoking status and physical exercise), and history of disease (T2DM, hypertension, chronic kidney disease (CKD), cancer, and history of fracture) were considered as covariates in the present study. PIR was calculated as the ratio of the midpoint of the household’s self-reported income to the corresponding poverty threshold for the household [30]. PIR values below 1 indicate poverty, while PIR values of 1 to 3 and above 3 reflect relatively higher socioeconomic status [31]. Based on serum cotinine levels, we defined three categories of (i) non-smoker (<1.0 ng/mL); (ii) environmental tobacco smoke (ETS) exposure (1.0–9.9 ng/mL); (iii) current smoker (≥10 ng/mL) [32]. Following the WHO guidelines, we defined four physical activity categories as: (i) inactive (participants with no regular physical activity); (ii) insufficient (<8.33 MET-hours/week); (iii) moderate (8.33–16.67 MET-hours/week); (iv) high (>16.67 MET-hours/week) [33]. Participants were diagnosed with T2DM if they satisfied any of the following criteria: (i) self-reported doctor diagnosis of diabetes or treatment with hypoglycemic drugs; (ii) fasting plasma glucose (FPG) of ≥7.0 mmol/L; (iii) 2-h blood glucose after oral glucose tolerance test (OGTT) of ≥11.1 mmol/L; (iv) hemoglobin A1c (HbA1c) of ≥6.5%; (v) any one of three random blood glucose test results ≥11.1 mmol/L [34]. CKD was diagnosed if participants met any of the following criteria: (i) estimated glomerular filtration rate (eGFR) of <60 mL/min/1.73m^2^; (ii) albumin-to-creatinine ratio (ACR) of >30 mg/g [35]. Hypertension was considered present if participants met either of the following criteria: (i) self-reported physician-diagnosed hypertension or treatment with anti-hypertensive medication; (ii) average of three systolic blood pressures (SBP) of ≥140 mmHg or average of three diastolic blood pressures (DBP) of ≥90 mmHg. Covariate data for cancer and history of fracture were obtained from the respective questionnaires administered to the study participants.

### 2.5. Statistical Analysis

All analyses used sampling weights recommended by the NCHS to account for the complex NHANES survey design. Initially, categorical variables were described by the frequency (percentage) of participants, and the differences between groups were compared using the chi-square test. Secondly, we measured the associations between the three plant-based diet indexes and the BMD T score, using Spearman’s correlation coefficients, and induced the corresponding 95% confidence intervals. Correlation coefficients were classified into five categories: very strong (0.90–1.00), strong (0.70–0.89), moderate (0.40–0.69), weak (0.10–0.39), and negligible (0–0.10) [36]. A significance test was necessary to control for the possibility that an observed difference between two correlations may be due to chance alone. Overlapping correlations in dependent groups were compared using Hittner, May, and Silver’s modification of Dunn and Clark’s z test and Zou’s confidence interval test [37]. In addition, multinomial logistic regression analysis was applied to examine the relationship between the three plant-based diet indexes and different BMD statuses. We developed two separate models for the association between each plant-based diet index and different BMD status: (1) Model 1: adjusted for age, sex, and ethnicity; (2) Model 2: Model 1 plus education, marital status, PIR, BMI, smoking status, physical exercise, hypertension, T2DM, CKD, cancer, and history of fracture. In the fully adjusted model (Model 2), we also explored the independent relationships of 15 individual food items with different BMD statuses.

We further conducted sensitivity analyses to evaluate the robustness of our findings. First, subgroup analyses were performed for variables associated with different BMD statuses, with stratification factors including age (20–50, 50–65, ≥65), sex (male, female), ethnicity (non-Hispanic black, non-Hispanic white, Mexican American, other), T2DM (yes, no), CKD (yes, no), history of fracture (yes, no), and smoking status (non-smoker, current smoker). Second, we excluded participants who had previously taken anti-osteoporotic and estrogenic drugs, as the use of these medications may affect the accuracy of the results. Then, we additionally adjusted the models for more potential confounders, including menopausal status, corticosteroid usage, and dietary supplements (vitamin D and calcium). Next, by calculating E values we evaluated the possibility of unmeasured confounding between the three plant-based dietary indexes and bone loss [38,39]. The E value estimates the required magnitude of an unmeasured confounding that could negate the observed association between the three plant-based dietary indexes and different BMD statuses.

All statistical tests were two-tailed, and a statistically significant difference was defined as *p* < 0.05. All analyses were performed using R software (4.1.0, R core team).

## 3. Results

### 3.1. Characteristics of Participants

A total of 16,085 participants were included in this study, among whom 8238 (51.22%) were female, and 4631 (28.79%) were over 65 years of age, regardless of gender. The characteristics of the study population are shown in Table 1, categorized for the groups with normal BMD, osteopenia, and osteoporosis. The three groups significantly differed in age, sex, ethnicity, education, marital status, PIR, BMI, smoking status, physical exercise, hypertension, T2DM, CKD, cancer, history of fracture, hPDI, PDI, and uPDI (*p* < 0.05).

### 3.2. Correlation between Plant-Based Diet Indexes and BMD T-Score

**Figure 2:**
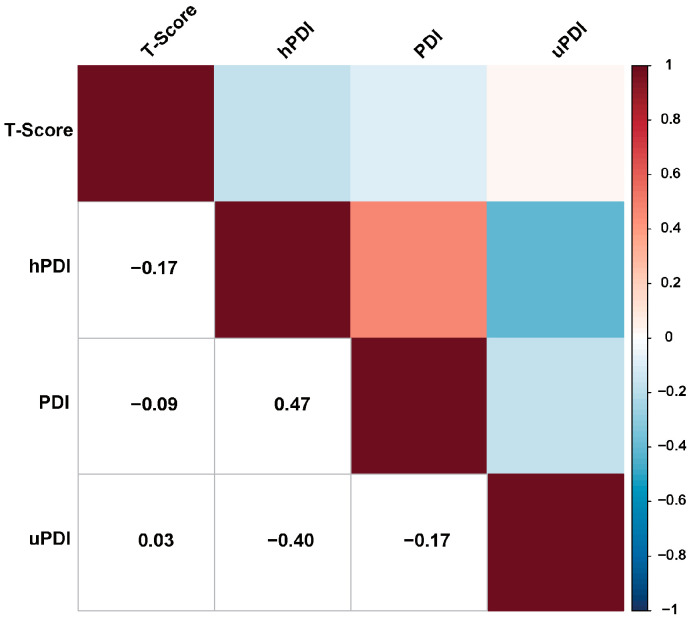
Intercorrelations between three plant-based diet indexes and BMD T-score. Abbreviations: PDI, plant-based diet index; hPDI, healthy plant-based diet index; uPDI, unhealthy plant-based diet index.

In the current study, we employed correlation analysis to assess the strength of the association between plant-based dietary indexes and BMD T scores. The Spearman correlation coefficients of the three plant-based dietary indexes and BMD T scores are illustrated in Figure 2 and Supplementary Table S2. hPDI was negatively and weakly correlated with BMD T score (r = −0.17; *p* < 0.001), while PDI (r = −0.09) and uPDI (r = 0.03) each had a negligible correlation with BMD T score. Furthermore, we conducted a comparative analysis of these associations and observed a significantly stronger correlation between hPDI and BMD T score in comparison with the other two associations (r (T score, hPDI) vs. r (T score, PDI): *p*-value < 0.001 (Hittner2003); 95% CI: −0.10; −0.06 (Zou2007); r (T score, hPDI) vs. r (T score, uPDI): *p*-value < 0.011 (Hittner2003); 95% CI: −0.23; −0.17 (Zou2007)). See Supplementary Table S3 for the detailed results of the correlation comparisons.

### 3.3. Associations between Plant-Based Diet Indexes and Different BMD Status Groups

We considered the association between three plant-based diet indexes and different BMD status groups, using multinomial logistic regression. Table 2 presents the logistic regression results (OR with 95% CI) and reports the results of a linear trend test (P for trend) to examine whether there was a linear trend in the association between bone loss and variations in three plant-based diet indexes. In the fully adjusted model, participants in the highest quintile for both hPDI (OR_Q5 vs. Q1_ = 1.50; 95% CI: 1.24–1.81) and PDI (OR_Q5 vs. Q1_ = 1.22; 95% CI: 1.03–1.45) had a positive association with osteopenia compared with participants in the lowest quintile, while the highest uPDI (OR_Q5 vs. Q1_ = 1.48; 95% CI: 1.04–2.11) was positively associated with osteoporosis. Furthermore, we found a positive association between a 10-unit increment in hPDI (OR_per 10-unit increment_ = 1.17; 95% CI: 1.08–1.27) and osteopenia, while a 10-unit increment in uPDI (OR_per 10-unit increment_ = 1.29; 95% CI: 1.08–1.54) was positively associated with osteoporosis.

We further compared the differences in the association between the three plant-based dietary indexes and different BMD statuses in the femoral neck and lumbar spine (Table 3). For the lumbar spine, hPDI (OR_Q5 vs. Q1_ = 1.25; 95% CI: 1.03–1.51; OR_per 10-unit increment_ = 1.11; 95% CI: 1.02–1.21) and PDI (OR_Q5 vs. Q1_ = 1.33; 95% CI: 1.07–1.67; OR_per 10-unit increment_ = 1.22; 95% CI: 1.03–1.45) were also proved to be risk factors for osteopenia, while uPDI was found to be a risk factor for both osteopenia (OR_Q2 vs. Q1_ = 1.23; 95% CI: 1.01–1.50) and osteoporosis (OR_Q5 vs. Q1_ = 1.58; 95% CI: 1.04–2.39; OR_per 10-unit increment_ = 1.31; 95% CI: 1.09–1.58). For the femoral neck, however, only uPDI was a risk factor for both osteopenia (OR_Q4 vs. Q1_ = 1.26; 95% CI: 1.03–1.56) and osteoporosis (OR_Q5 vs. Q1_ = 2.27; 95% CI: 1.03–5.02; OR_per 10-unit increment_ = 1.92; 95% CI: 1.47–2.51), with no significant association between the remaining two plant-based dietary indexes and bone loss.

### 3.4. Associations between 15 Individual Food Items and Different BMD Status Groups

We analyzed the independent associations of 15 individual food items with different BMD statuses. Table 4 shows the significant results of multiple logistic regression analysis using six individual food items (vegetables, nuts, refined grain, animal fat, eggs, meat) in association with bone loss. For individual plant food items, participants who consumed more nuts (OR_Q5 vs. Q1_= 1.22; 95% CI: 1.01–1.46) had higher odds of osteopenia, while participants who consumed more refined grain (OR_Q5 vs. Q1_= 0.73; 95% CI: 0.61–0.87) and vegetables (OR_Q5 vs. Q1_= 0.64; 95% CI: 0.43–0.95) had lower odds of osteopenia and osteoporosis, respectively. For individual animal food items, participants who consumed more animal fat (OR_Q5 vs. Q1 (osteopenia vs. normal)_ = 0.81; 95% CI: 0.66–0.99; OR_Q5 vs. Q1 (osteoporosis vs. normal)_ = 0.57; 95% CI: 0.39–0.83), eggs (OR_Q3 vs. Q1 (osteopenia vs. normal)_ = 0.79; 95% CI: 0.65–0.96; OR_Q3 vs. Q1 (osteoporosis vs. normal)_ = 0.66; 95% CI: 0.47–0.93), and meat (OR_Q4 vs. Q1 (osteopenia vs. normal)_ = 0.77; 95% CI: 0.65–0.92; OR_Q5 vs. Q1 (osteoporosis vs. normal)_ = 0.64; 95% CI: 0.44–0.93) had lower odds of osteopenia and osteoporosis.

### 3.5. Sensitivity Analysis

**Figure 3:**
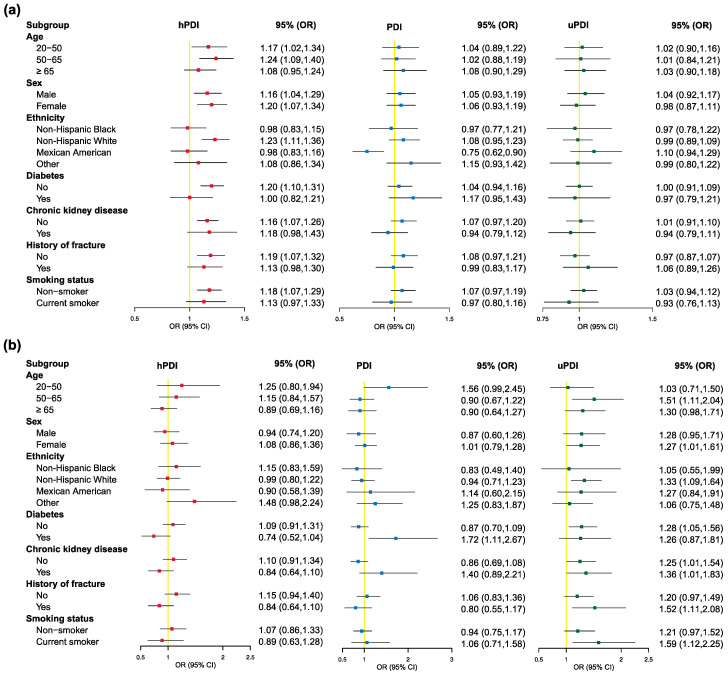
(**a**) ORs and 95% CI for osteopenia per 10-unit increment in adherence to plant-based diet indexes, stratified by selected characteristics; (**b**) ORs and 95% CI for osteoporosis per 10-unit increment in adherence to plant-based diet indexes, stratified by selected characteristics. All models were multivariable adjusted for age, sex, and ethnicity, education, marital status, PIR, BMI, smoking status, physical exercise, hypertension, T2DM, CKD, cancer, and history of fracture. In each stratified analysis, the stratification variable was excluded in the adjustments. The red dots presented hPDI; the blue dots presented PDI; the green dots presented uPDI.

Multiple sensitivity analyses were conducted to assess the robustness of our results. Firstly, subgroup analysis suggested a consistent association between plant-based dietary indexes and osteopenia/osteoporosis across most subgroups. The positive association between osteopenia and a 10-unit increment in hPDI was observed in strata defined by age (20–50 and 50–65), sex (male and female), ethnicity (non-Hispanic white), T2DM (no), CKD (no), fracture history (no), and smoking status (non-smoker) (Figure 3a). Also, we found a positive association between a 10-unit increment in uPDI and osteoporosis manifested in participants with the following characteristics: age from 50 to 65 years, female, non-Hispanic white, non-diabetes, fracture history, and current smoker (Figure 3b). Further detailed results are listed in Figure 3a,b.

Secondly, the results remained robust and significant after excluding participants with a history of taking anti-osteoporosis and estrogenic drugs. Further detailed results are listed in Supplementary Table S4.

Moreover, after further adjustment for three potential confounders (menopausal status, corticosteroid usage, and dietary supplements), respectively, the results remained robust and significant (Supplementary Tables S5–S7).

Finally, we calculated E values to evaluate the sensitivity of our findings to potential unmeasured confounding factors. As shown in Supplementary Table S8, the E values indicated that our results were robust unless there was an unmeasured confounding factor with a relative risk greater than the E values that were associated with the three plant-based dietary indexes.

## 4. Discussion

This study assessed the association between three plant-based dietary indexes and osteopenia/osteoporosis, based on a cross-sectional study of the adult population in the United States. According to the correlation coefficient analysis, a close association was observed between hPDI and BMD T score. Moreover, we observed a harmful association between PDI and hPDI with osteopenia, and a harmful association between uPDI and osteoporosis. Meanwhile, higher hPDI and PDI were significantly correlated with higher risk of osteopenia at the lumbar spine but not at the femoral neck. Among individual food items, vegetables, refined grains, animal fats, eggs, and meat were the main protective contributors, while nuts were associated with higher odds of osteopenia.

Several previous studies have indicated that a plant-based diet may have a negative impact on bone health, which is consistent with our findings. For instance, a Chinese cohort study reported significant differences in hPDI between three groups (normal, osteopenia, and osteoporosis), with the normal group having a significantly lower hPDI than the osteopenia and osteoporosis groups [27]. In an Iranian case–control study of post-menopausal women with osteoporosis, a higher uPDI was associated with an increased risk of bone loss in the femoral neck (OR: 2.63; 95% CI: 1.37–5.06) and lumbar spine (OR: 4.23; 95% CI: 2.19–8.19) [26]. Furthermore, several meta-analyses have compared the impact of plant-based and omnivorous diets on bone health [40,41]. Li et al. concluded that individuals following a plant-based diet had significantly lower BMD at the lumbar spine, femoral neck, and whole body compared to those following an omnivorous diet, with a reduction of approximately 3–4% [40]. Another meta-analysis found that vegans had 6% lower BMD compared with omnivores [41]. Taken together, our findings provide further evidence supporting the potential harmful association between bone health and a plant-based diet.

Several potential mechanisms have been proposed to explain the association between a plant-based diet and an increased risk of bone loss. A clinical trial reported that a plant-based diet reduced calcium and vitamin D intake and increased N-tetrapeptide biomarkers, indicating increased bone resorption [42]. Furthermore, a cross-sectional study of Iranian older adults showed that higher uPDI was significantly associated with decreased levels of osteocalcin, a protein that plays an critical role in regulating bone formation and remodeling by modulating bone mineralization and the activity of osteoblasts and osteoclasts [8,43]. Moreover, individuals following a plant-based diet typically have lower dietary calcium and protein intake than omnivores [44,45]. Adequate protein intake is crucial for the formation and maintenance of the bone matrix and influences the secretion of insulin-like growth factor I (IGF-I), an orthotropic hormone that promotes calcium and phosphorus absorption in the gut and synthesizes calcitriol [16]. However, the relationship between protein and dietary calcium is complex and interdependent [41]. While protein has positive effects on bone health, adequate dietary calcium intake is also necessary. Inadequate calcium levels may lead to adverse affects of protein on bone density [16]. Therefore, individuals following a plant-based diet require additional sources of protein and dietary calcium. For individual foods, we found beneficial associations between bone health and vegetables, eggs, and meat, which are high-quality dietary calcium and protein sources. We recommend that individuals following a plant-based diet carefully select appropriate foods or supplements to enhance their calcium and protein intake, ensuring adequate intake to avoid potential nutrient shortfalls and to help maintain a healthy bone balance.

It is worth noting that our study revealed that an overall plant-based diet and a healthy plant-based diet affect osteopenia rather than osteoporosis. Bone metabolism and BMD are sensitive to subtle changes in nutrient intake and acid–base balance [46]. While several studies and potential mechanisms described above suggest that a plant-based diet can negatively affect bone health, it is crucial to note that plant-based diets also possess potential benefits for bone health. Higher PDI and hPDI, compared with uPDI, may reflect higher intake of whole grains, vegetables, fruits, nuts, legumes, tea, and coffee. These healthy plant foods typically contain higher levels of minerals (magnesium, potassium) and vitamins, as well as antioxidant and anti-inflammatory phytochemicals. Consequently, these foods result in a lower acid load on the body, which is beneficial for bone health [47]. For instance, fresh fruits and vegetables are rich in potassium, which can reduce the acid load to maintain calcium levels in bones [26]. The positive effects of a plant-based diet on bone health may counteract some of the negative effects and thus attenuate the negative impact on BMD, which may potentially explain our findings. Therefore, future public health recommendations should focus on both the quantity and quality of plant-based foods in order to maintain optimal bone health.

The prevalence of osteoporosis varies widely within the body, with the lumbar spine and femoral neck being the most common sites for diagnosing osteoporosis [48]. The findings of the present study indicate a significant association between plant-based dietary indexes (hPDI and PDI) and osteopenia at the lumbar spine, whereas no significant association was observed at the femoral neck. There are several potential explanations for these results. Firstly, the lumbar spine and femoral neck have different rates of bone loss, with trabecular bone (in the lumbar spine) having a more rapid rate of bone loss than cortical bone (in the femur) [49,50]. Secondly, most etiologies of secondary osteoporosis, such as malabsorption, liver disease, and rheumatoid arthritis, affect the spine rather than the femur [2,51]. Moreover, weight bearing, particularly in the hip and femur, is known to increase BMD and is a cause of physiological variation [2].

The subgroup analysis findings suggest that certain groups may experience more pronounced effects under plant-based dietary patterns, such as non-Hispanic white populations and relatively young individuals. To provide a comprehensive explanation for the observed variations in bone health across ethnicities, it is imperative to consider the influence of genetic factors on BMD. Genomic studies indicate that disparities in BMD across ethnicities may primarily be attributed to genetic dissimilarities [52]. Earlier investigations established that the incidence of osteoporosis significantly differs between ethnic groups, with more than 60.0% of the variance in BMD being attributed to genetic factors [53]. Additionally, genetic factors affect differences in body composition, such as greater cortical thickness and higher trabecular BMD in blacks, suggest inherent differences in bone strength according to ethnicity [54]. Several potential explanations could be considered regarding the observed differences in the effects of plant-based diets on bone health among distinct demographic strata defined by age. For example, younger individuals may have higher rates of bone turnover and may require more nutrients, such as calcium and vitamin D, to maintain bone health [55]. Additionally, older individuals may be at higher risk of osteoporosis and may require more targeted interventions to maintain bone health [2]. However, it is important to note that the complex interplay of these factors with ethnicity and age is not clear, and further studies are needed to fully elucidate their underlying impact on bone health.

To the best of our knowledge, there remains relatively scarce research assessing the association between plant-based dietary indexes and bone loss, especially in the US population. Our study offers timely and unique evidence with important implications. Given the increasing recommendation of plant-based diets for environmental protection and prevention of chronic disease, it is necessary to investigate their relationship with bone health. Our findings may contribute to future nutritional policy development and promote systemic and synergistic shifts toward a healthier food system. The present study has several notable strengths. First, the relatively large sample size in NHANES and the use of population-based random cluster selection ensured the representativeness of the sample and its applicability to the entire U.S. population. Second, the design of the subgroup and sensitivity analyses enabled a more in-depth exploration of the association between plant-based dietary indexes and bone health, strengthening the reliability of the study results. Taken together, these strengths contribute to the validity and generalizability of our findings. However, there are several limitations that need to be cautiously considered when interpreting our findings. First, the present study employed a cross-sectional design, precluding us from establishing a causal relationship between plant-based dietary indexes and osteopenia/osteoporosis. Second, relying on 24-h dietary recalls to collect the dietary data may not accurately reflect participants’ usual dietary intake. Finally, since the environment surveyed by NHANES is restricted to the USA, the generalizability of the findings to other dietary cultures may be limited, and further research remains necessary.

## 5. Conclusions

In conclusion, our findings provide evidence that adherence to a plant-based dietary pattern is associated with decreased BMD in a nationally representative population of US adults, highlighting the importance of a balanced diet for maintaining bone health, especially including foods rich in dietary calcium and protein such as vegetables, eggs, and meat. Meanwhile, a negative association was revealed between two plant-based dietary indexes (hPDI and PDI) and osteopenia, which was more significantly at the lumbar spine rather than the femoral neck. Among 15 individual food items, vegetables, refined grains, animal fat, eggs, and meat were the main protective contributors, whereas nuts were associated with increased odds of osteopenia. From a clinical perspective, dietary interventions rather than medications may be more effective in improving bone health and preventing fractures. Individuals following a plant-based diet should carefully plan their nutritional intake and monitor their bone health regularly. Moreover, further research is needed to explore the causality and generalizability of our findings, as well as to investigate the potential benefits and risks of specific types of plant-based diets in terms of their effects on bone health.

## Figures and Tables

**Table 1:** Baseline characteristics of the study participants.

Characteristic	Overall (N = 16,085)	Normal (N = 9608)	Osteopenia (N = 5404)	Osteoporosis (N = 1073)	*p*-Value
**Demographic characteristics**					
Age, n (no. weighted %)					**<0.001**
20–50	6377 (39.65)	4775 (54.66)	1501 (31.43)	101 (11.09)	
50–65	5077 (31.56)	2889 (30.71)	1839 (38.31)	349 (33.60)	
≥65	4631 (28.79)	1944 (14.63)	2064 (30.26)	623 (55.31)	
Sex, n (no. weighted %)					**<0.001**
Female	8238 (51.22)	4259 (45.19)	3152 (60.87)	827 (80.21)	
Male	7847 (48.78)	5349 (54.81)	2252 (39.13)	246 (19.79)	
Ethnicity, n (no. weighted %)					**<0.001**
Non-Hispanic black	3163 (19.66)	2389 (13.34)	687 (5.99)	87 (4.39)	
Mexican American	2614 (16.26)	1569 (7.94)	863 (7.02)	182 (6.00)	
Non-Hispanic white	7844 (48.77)	4329 (68.69)	2936 (75.54)	579 (76.79)	
Other	2464 (15.32)	1321 (10.03)	918 (11.45)	225 (12.82)	
Education, n (no. weighted %)					**<0.001**
Less than high school	6710 (41.76)	3921 (32.53)	2277 (32.02)	512 (35.85)	
High school	1074 (6.68)	561 (5.93)	401 (8.60)	112 (12.57)	
More than high school	8283 (51.55)	5118 (61.54)	2717 (59.38)	448 (51.58)	
PIR, n (no. weighted %)					**0.002**
<1	2665 (17.95)	1569 (11.87)	896 (11.36)	200 (13.72)	
1–3	6156 (41.47)	3580 (33.00)	2115 (34.69)	461 (42.84)	
≥3	6024 (40.58)	3751 (55.13)	1966 (53.95)	307 (43.45)	
BMI, n (no. weighted %)					**<0.001**
<25	4453 (27.78)	1936 (22.70)	1968 (39.00)	549 (54.02)	
25–30	5709 (35.62)	3414 (35.01)	1957 (34.88)	338 (29.80)	
≥30	5865 (36.59)	4227 (42.29)	1456 (26.13)	182 (16.18)	
**Lifestyle characteristics**					
Smoking status, n (no. weighted %)					**<0.001**
Non-smoker	11,356 (73.12)	6561 (71.04)	3991 (76.12)	804 (76.49)	
ETS	550 (3.54)	381 (3.65)	149 (2.78)	20 (1.47)	
Current smoker	3624 (23.34)	2309 (25.31)	1096 (21.09)	219 (22.04)	
Marital status, n (no. weighted %)					**<0.001**
Married/cohabiting	10,075 (62.67)	6171 (67.44)	3342 (65.16)	562 (53.29)	
Widowed/divorced/separated	3886 (24.17)	1909 (16.43)	1533 (24.11)	444 (40.28)	
Never married	2115 (13.16)	1521 (16.12)	528 (10.73)	66 (6.43)	
Physical exercise, n (no. weighted %)					**<0.001**
Inactive	4249 (26.42)	2275 (19.36)	1552 (22.24)	422 (33.18)	
Insufficient	2895 (18.00)	1734 (18.99)	972 (19.75)	189 (17.54)	
Moderate	1983 (12.33)	1171 (12.62)	683 (14.24)	129 (15.31)	
High	6958 (43.26)	4428 (49.03)	2197 (43.78)	333 (33.97)	
**History of disease**					
T2DM, n (no. weighted %)					**0.01**
No	12,907 (80.24)	7668 (84.55)	4390 (87.10)	849 (84.33)	
Yes	3178 (19.76)	1940 (15.45)	1014 (12.90)	224 (15.67)	
Hypertension, n (no. weighted %)					**<0.001**
No	8591 (53.41)	5379 (60.71)	2758 (56.57)	454 (45.15)	
Yes	7494 (46.59)	4229 (39.29)	2646 (43.43)	619 (54.85)	
CKD, n (no. weighted %)					**<0.001**
No	12,428 (80.17)	7670 (86.66)	4055 (82.60)	703 (71.45)	
Yes	3074 (19.83)	1573 (13.34)	1170 (17.40)	331 (28.55)	
Cancer, n (no. weighted %)					**<0.001**
No	14,228 (88.53)	8696 (90.88)	4646 (85.55)	886 (81.72)	
Yes	1844 (11.47)	902 (9.12)	755 (14.45)	187 (18.28)	
History of fracture, n (no. weighted %)					**<0.001**
No	12,359 (76.91)	7624 (77.87)	3981 (72.62)	754 (66.63)	
Yes	3711 (23.09)	1975 (22.13)	1417 (27.38)	319 (33.37)	
**Plant-based diet index**					
hPDI, n (no. weighted %)					**<0.001**
Q1 (22–39)	3635 (22.60)	2561 (26.90)	937 (16.41)	137 (13.15)	
Q2 (40–43)	3046 (18.94)	1922 (20.19)	972 (18.21)	152 (13.66)	
Q3 (44–47)	3395 (21.11)	1983 (20.58)	1159 (20.79)	253 (22.51)	
Q4 (48–51)	2874 (17.87)	1573 (16.06)	1052 (19.35)	249 (23.60)	
Q5 (52–69)	3135 (19.49)	1569 (16.27)	1284 (25.24)	282 (27.07)	
PDI, n (no. weighted %)					**<0.001**
Q1 (23–39)	3697 (22.98)	2392 (24.39)	1117 (18.86)	188 (17.10)	
Q2 (40–42)	3139 (19.52)	1962 (20.45)	996 (17.90)	181 (16.29)	
Q3 (43–45)	3403 (21.16)	2011 (22.22)	1125 (22.03)	267 (23.92)	
Q4 (46–48)	2824 (17.56)	1611 (16.39)	995 (18.40)	218 (19.93)	
Q5 (49–65)	3022 (18.79)	1632 (16.55)	1171 (22.81)	219 (22.76)	
uPDI, n (no. weighted %)					**0.01**
Q1 (24–42)	3538 (22.00)	2092 (25.25)	1223 (26.98)	223 (23.57)	
Q2 (43–46)	3582 (22.27)	2085 (22.08)	1264 (23.39)	233 (22.42)	
Q3 (47–49)	3005 (18.86)	1726 (17.91)	1064 (19.32)	215 (20.10)	
Q4 (50–53)	3388 (21.06)	2048 (19.65)	1101 (18.55)	239 (19.71)	
Q5 (54–68)	2572 (15.99)	1657 (15.12)	752 (11.75)	163 (14.20)	

Missing rates were 0.06% for marital status, 7.71% for PIR, 0.11% for education, 0.36% for BMI, 3.45% for smoking status, 3.62% for CKD, 0.08% for cancer, 0.09% for history of fracture. Abbreviations: PIR, poverty income ratio; BMI, body mass index; CKD, chronic kidney disease; PDI, plant-based diet index; hPDI, healthy plant-based diet index; uPDI, unhealthy plant-based diet index.

**Table 2:** ORs and 95% CIs for bone loss, according to quintiles for hPDI, PDI, and uPDI.

	Osteopenia vs. Normal		Osteoporosis vs. Normal	
	Model 1 OR (95% CI)	Model 2 OR (95% CI)	Model 1 OR (95% CI)	Model 2 OR (95% CI)
**hPDI**				
Q1	1 (Reference)	1 (Reference)	1 (Reference)	1 (Reference)
Q2	**1.18 (1.01, 1.39)**	**1.24 (1.04, 1.48)**	0.83 (0.55, 1.27)	0.89 (0.56, 1.41)
Q3	1.19 (0.99, 1.44)	**1.21 (1.00, 1.47)**	1.06 (0.73, 1.53)	0.95 (0.65, 1.40)
Q4	**1.23 (1.03, 1.46)**	**1.24 (1.02, 1.51)**	1.06 (0.74, 1.51)	1.02 (0.71, 1.46)
Q5	**1.49 (1.27, 1.75)**	**1.50 (1.24, 1.81)**	1.06 (0.72, 1.55)	1.08 (0.73, 1.60)
Per 10-unit increment	**1.18 (1.10, 1.26)**	**1.17 (1.08, 1.27)**	1.04 (0.89, 1.22)	1.03 (0.88, 1.21)
*p* for trend	**<0.001**	**<0.001**	0.51	0.49
**PDI**				
Q1	1 (Reference)	1 (Reference)	1 (Reference)	1 (Reference)
Q2	1.00 (0.84, 1.20)	0.93 (0.77,1.13)	0.87 (0.60,1.26)	0.80 (0.53, 1.22)
Q3	1.08 (0.90, 1.30)	1.00 (0.82,1.21)	1.09 (0.76,1.57)	1.00 (0.67, 1.49)
Q4	1.20 (1.00, 1.45)	1.14 (0.93,1.38)	1.15 (0.80,1.65)	1.09 (0.71, 1.69)
Q5	**1.36 (1.19, 1.56)**	**1.22 (1.03,1.45)**	1.16 (0.81,1.66)	1.01 (0.68, 1.51)
Per 10-unit increment	**1.13 (1.05, 1.21)**	1.06 (0.96,1.16)	1.05 (0.88,1.25)	0.97 (0.79, 1.19)
*p* for trend	**<0.001**	**0.003**	0.21	0.53
**uPDI**				
Q1	1 (Reference)	1 (Reference)	1 (Reference)	1 (Reference)
Q2	1.03 (0.91, 1.17)	1.05 (0.90, 1.21)	1.09 (0.79, 1.49)	1.03 (0.71, 1.50)
Q3	1.13 (0.97, 1.32)	1.15 (0.97, 1.37)	**1.41 (1.01, 1.97)**	1.27 (0.87, 1.86)
Q4	1.07 (0.94, 1.23)	1.05 (0.91, 1.22)	**1.48 (1.09, 2.03)**	1.39 (0.98, 1.98)
Q5	1.02 (0.86, 1.19)	0.97 (0.79, 1.20)	**1.78 (1.32, 2.40)**	**1.48 (1.04, 2.11)**
Per 10-unit increment	1.02 (0.96, 1.09)	1.00 (0.92, 1.08)	**1.37 (1.16, 1.61)**	**1.29 (1.08, 1.54)**
*p* for trend	0.48	0.89	**<0.001**	**0.01**

Note: Model 1: adjusted for age, sex, and ethnicity; Model 2: Model 1 plus education, marital status, PIR, BMI, smoking status, physical exercise, hypertension, T2DM, CKD, cancer, and history of fracture; *p* trends below 0.001 are presented as <0.001; a statistically significant difference was defined as *p* < 0.05 and data with *p* values below 0.05 are presented in bold type. Abbreviations: OR, odds ratio; 95% CI, 95% confidence interval; PDI, plant-based diet index; hPDI, healthy plant-based diet index; uPDI, unhealthy plant-based diet index.

**Table 3:** ORs and 95% CIs for bone loss at the femoral neck and lumbar spine, according to quintiles for hPDI, PDI, and uPDI.

	Femoral Neck		Lumbar Spine	
	Osteopenia vs. Normal	Osteoporosis vs. Normal	Osteopenia vs. Normal	Osteoporosis vs. Normal
**hPDI**				
Q1	1 (Reference)	1 (Reference)	1 (Reference)	1 (Reference)
Q2	1.25 (0.95, 1.65)	1.09 (0.51, 2.33)	0.97 (0.79, 1.20)	0.59 (0.34, 1.03)
Q3	1.23 (0.94, 1.61)	0.68 (0.32, 1.43)	1.18 (0.95, 1.46)	0.80 (0.47, 1.38)
Q4	1.20 (0.90, 1.60)	1.23 (0.66, 2.29)	1.18 (0.98, 1.42)	1.01 (0.58, 1.74)
Q5	1.27 (0.98, 1.63)	0.74 (0.37, 1.47)	**1.25 (1.03, 1.51)**	1.08 (0.67, 1.74)
Per 10-unit increment	1.07 (0.96, 1.19)	0.88 (0.68, 1.12)	**1.11 (1.02, 1.21)**	1.07 (0.88, 1.31)
*p* for trend	0.12	0.53	**0.01**	0.21
**PDI**				
Q1	1 (Reference)	1 (Reference)	1 (Reference)	1 (Reference)
Q2	0.81 (0.61, 1.08)	0.58 (0.27, 1.28)	0.98 (0.77, 1.25)	0.86 (0.55, 1.34)
Q3	0.90 (0.68, 1.19)	0.86 (0.42, 1.75)	0.96 (0.76, 1.22)	1.09 (0.71, 1.67)
Q4	0.89 (0.68, 1.15)	0.75 (0.38, 1.50)	1.21 (0.94, 1.55)	1.42 (0.97, 2.06)
Q5	0.99 (0.77, 1.28)	0.69 (0.34, 1.39)	**1.33 (1.07, 1.67)**	1.19 (0.76, 1.88)
Per 10-unit increment	0.94 (0.81, 1.08)	0.78 (0.56, 1.10)	**1.15 (1.02, 1.28)**	1.13 (0.90, 1.40)
*p* for trend	0.84	0.45	**0.003**	0.13
**uPDI**				
Q1	1 (Reference)	1 (Reference)	1 (Reference)	1 (Reference)
Q2	1.13 (0.93, 1.38)	0.86 (0.50, 1.48)	**1.23 (1.01, 1.50)**	**1.60 (1.04, 2.45)**
Q3	1.10 (0.67, 2.18)	1.20 (0.92, 2.01)	1.04 (0.83, 1.30)	1.27 (0.82, 1.98)
Q4	**1.26 (1.03, 1.56)**	**2.17 (1.25, 3.79)**	1.09 (0.90, 1.33)	**1.92 (1.24, 2.98)**
Q5	1.15 (0.89, 1.49)	**2.27 (1.03, 5.02)**	1.14 (0.93, 1.41)	**1.58 (1.04, 2.39)**
Per 10-unit increment	1.09 (0.97, 1.22)	**1.92 (1.47, 2.51)**	1.03 (0.93, 1.14)	**1.31 (1.09, 1.58)**
*p* for trend	0.12	**0.01**	0.40	**0.007**

Note: Fully adjusted model: age, sex, and ethnicity, education, marital status, PIR, BMI, smoking status, physical exercise, hypertension, T2DM, CKD, cancer, and history of fracture; a statistically significant difference was defined as *p* < 0.05 and data with *p* values below 0.05 are presented in bold type. Abbreviations: OR, odds ratio; 95% CI, 95% confidence interval; PDI, plant-based diet index; hPDI, healthy plant-based diet index; uPDI, unhealthy plant-based diet index.

**Table 4:** ORs and 95% CIs for bone loss, according to individual food groups.

Groups	Osteopenia vs. Normal	*p* for Trend	Osteoporosis vs. Normal	*p* for Trend
Vegetables		0.40		**0.02**
Q1	1 (Reference)		1 (Reference)	
Q2	0.86 (0.72, 1.04)		0.98 (0.74, 1.30)	
Q3	0.99 (0.82, 1.19)		0.83 (0.59, 1.15)	
Q4	0.99 (0.81, 1.20)		0.81 (0.57, 1.14)	
Q5	1.00 (0.84, 1.20)		**0.64 (0.43, 0.95)**	
Nuts		**0.01**		0.85
Q1	1 (Reference)		1 (Reference)	
Q2	0.99 (0.85, 1.15)		0.96 (0.69, 1.35)	
Q3	1.02 (0.84, 1.24)		0.86 (0.56, 1.31)	
Q4	1.19 (0.99, 1.43)		1.20 (0.82, 1.75)	
Q5	**1.22 (1.01, 1.46)**		0.94 (0.66, 1.35)	
Refined grain		**<0.001**		0.63
Q1	1 (Reference)		1 (Reference)	
Q2	0.88 (0.74, 1.04)		1.17 (0.82, 1.66)	
Q3	0.86 (0.73, 1.01)		1.05 (0.76, 1.46)	
Q4	**0.82 (0.69, 0.98)**		0.86 (0.59, 1.25)	
Q5	**0.73 (0.61, 0.87)**		1.29 (0.82, 2.03)	
Animal fat		**0.01**		**0.004**
Q1	1 (Reference)		1 (Reference)	
Q2	0.96 (0.79, 1.17)		0.88 (0.61, 1.25)	
Q3	0.96 (0.78, 1.17)		0.98 (0.72, 1.34)	
Q4	**0.80 (0.64, 0.99)**		0.82 (0.58, 1.16)	
Q5	**0.81 (0.66, 0.99)**		**0.57 (0.39, 0.83)**	
Eggs		0.22		0.67
Q1	1 (Reference)		1 (Reference)	
Q2	0.99 (0.82, 1.20)		0.70 (0.48, 1.01)	
Q3	**0.79 (0.65, 0.96)**		**0.66 (0.47, 0.93)**	
Q4	0.89 (0.77, 1.03)		0.82 (0.58, 1.16)	
Q5	0.88 (0.74, 1.04)		0.74 (0.50, 1.11)	
Meat		0.10		**0.001**
Q1	1 (Reference)		1 (Reference)	
Q2	0.89 (0.75, 1.06)		1.05 (0.74, 1.49)	
Q3	0.92 (0.76, 1.12)		1.02 (0.70, 1.47)	
Q4	**0.77 (0.65, 0.92)**		**0.58 (0.39, 0.85)**	
Q5	0.88 (0.73, 1.05)		**0.64 (0.44, 0.93)**	

ORs were adjusted for age, sex, and ethnicity, education, marital status, PIR, BMI, smoking status, physical exercise, hypertension, T2DM, CKD, cancer, and history of fracture; a statistically significant difference was defined as *p* < 0.05 and data with *p* values below 0.05 are presented in bold type.

## Data Availability

Publicly available datasets were analyzed in this study. This data can be found here: https://www.cdc.gov/nchs/nhanes/. It was accessed on 6 March 2023.

## Supplementary Materials

The following supporting information can be downloaded at: https://www.mdpi.com/article/10.3390/nu15071794/s1, Supplementary Table S1: Construction and scoring of plant-based diet indices; Supplementary Table S2: Spearman correlation coefficients and 95% confidence intervals between three plant-based diet indexes and BMD T score; Supplementary Table S3: Test results of comparisons of two overlapping correlations in dependent groups; Supplementary Table S4: ORs and 95% CIs for bone loss, excluding participants with baseline history of taking anti-osteoporosis or estrogenic drugs; Supplementary Table S5: ORs and 95% CIs for bone loss, additionally adjusted for menopausal status; Supplementary Table S6: ORs and 95% CIs for bone loss, additionally adjusted for corticosteroid usage; Supplementary Table S7: ORs and 95% CIs for bone loss, additionally adjusted for dietary supplements; Supplementary Table S8: E values for the effects of plant-based diets on bone loss (lower limit of 95% CI) in the fully adjusted model.

## Abbreviations

ACR	albumin-to-creatinine ratio;
BMD	bone mineral density;
BMI	body mass index;
CI	95% confidence interval
CKD	chronic kidney disease;
DBP	diastolic blood pressures;
eGFR	estimated glomerular filtration rate;
FPG	fasting plasma glucose;
HbA1c	hemoglobin A1c;
hPDI	healthy plant-based dietary index;
IGF-I	insulin-like growth factor I;
NCHS	National Center for Health Statistics;
NHANES	National Health and Nutrition Examination Survey;
OGTT	oral glucose tolerance test;
OR	odds ratio;
PDI	overall plant-based dietary index;
PIR	poverty income ratio;
SBP	systolic blood pressures;
T2DM	type 2 diabetes mellitus;
uPDI	unhealthy plant-based dietary index;
US	United States.
