# Supplementary material for: The Hidden Dangers of Plant-Based Diets Affecting Bone Health: A Cross-Sectional Study with U.S. National Health and Nutrition Examination Survey (NHANES) Data from 2005–2018
Source: Nutrients. 2023 Apr 6;15(7):1794. doi: 10.3390/nu15071794 (PMC10097387; doi:10.3390/nu15071794)
Supplement: Supplementary file 1 [file nutrients-15-01794-s001.zip › nutrients-2295223-supplementary.pdf]

nutrients

Supplementary Table S1. Construction and score of plant-based diet indices

	PDI	hPDI	uPDI
Plant-based Food Groups			
Healthy			
Whole grains	Postive scores	Postive scores	Reverse scores
Fruits	Postive scores	Postive scores	Reverse scores
Vegetables	Postive scores	Postive scores	Reverse scores
Nuts	Postive scores	Postive scores	Reverse scores
Legumes	Postive scores	Postive scores	Reverse scores
Tea and coffee	Postive scores	Postive scores	Reverse scores
Less healthy			
Fruit juices	Postive scores	Reverse scores	Postive scores
Refined grain	Postive scores	Reverse scores	Postive scores
Potatoes	Postive scores	Reverse scores	Postive scores
Sugar	Postive scores	Reverse scores	Postive scores
Animal-based Food Groups			
Animal fat	Reverse scores	Reverse scores	Reverse scores
Dairy	Reverse scores	Reverse scores	Reverse scores
Egg	Reverse scores	Reverse scores	Reverse scores
Seafood	Reverse scores	Reverse scores	Reverse scores
Meat	Reverse scores	Reverse scores	Reverse scores

Abbreviations: PDI, plant-based diet index; hPDI, healthful plant-based diet index; uPDI, unhealthful plant-based diet index.

Supplementary Table S2. Spearman correlation coefficients and 95% confidence intervals between three plant-based diet indexes and T-score.

	hPDI	PDI	uPDI
T-Score	**-0.17 (-0.19, -0.16)**	-0.09 (-0.11, -0.08)	0.03 (0.02, 0.05)

The T-score with the highest correlation coefficient for three plant-based diet indexes was marked in **bold.** Abbreviations: PDI, plant-based diet index; hPDI, healthful plant-based diet index; uPDI, unhealthful plant-based diet index. All are significant at the level of <0.001 (2-tailed).

Supplementary Table S3. Test results of comparisons of two overlapping correlations in dependent groups

	Hittner2003	Zou2007
	p-value	95% CI
r (T-Score, hPDI) vs. r (T-Score, PDI)	**<0.001**	**(-0.10, -0.06)**
r (T-Score, hPDI) vs. r (T-Score, uPDI)	**<0.001**	**(-0.23, -0.17)**

Hitter, May, and Silver's (2003) modification of Dunn and Clark's z (1969) using a back-transformed average Fisher's (1921) Z procedure, which is a significance test; Zou's (2007) confidence interval is a test based on the computation of confidence intervals. Results that reject the null hypothesis are marked in bold.

Supplementary Table S4. ORs and 95% CIs for bone loss, excluding participants with baseline history of taking anti-osteoporosis drugs and estrogenic drugs

	Osteopenia vs Normal OR (95% CI)	Osteoporosis vs Normal OR (95% CI)
hPDI		
Q1	1 (Reference)	1 (Reference)
Q2	**1.25 (1.04,1.50)**	0.84 (0.53,1.33)
Q3	1.19 (0.98,1.44)	0.82 (0.56,1.21)
Q4	**1.26 (1.03,1.54)**	1.13 (0.78,1.64)
Q5	**1.53 (1.26,1.87)**	1.03 (0.71,1.50)
Per 10-unit increment	**1.19 (1.09,1.29)**	1.04 (0.89,1.21)
PDI		
Q1	1 (Reference)	1 (Reference)
Q2	0.93 (0.78,1.12)	0.80 (0.52,1.24)
Q3	0.97 (0.80,1.18)	0.88 (0.58,1.35)
Q4	1.20 (0.99,1.45)	1.04 (0.68,1.60)
Q5	**1.24 (1.04,1.49)**	0.96 (0.60,1.52)
Per 10-unit increment	1.06 (0.97,1.17)	0.94 (0.75,1.18)
uPDI		
Q1	1 (Reference)	1 (Reference)
Q2	1.03 (0.88,1.19)	1.14 (0.75,1.72)
Q3	1.16 (0.97,1.39)	1.36 (0.92,2.01)
Q4	1.08 (0.92,1.27)	**1.52 (1.03,2.25)**
Q5	0.97 (0.78,1.20)	1.34 (0.95,1.88)
Per 10-unit increment	0.99 (0.89,1.09)	**1.23 (1.01,1.50)**

Note: Fully adjusted model: age, sex, and ethnicity, education, marital status, PIR, BMI, smoking status, physical exercise, hypertension, T2DM, CKD, cancer, and history of fracture. Abbreviations: OR, odds ratio; 95% CI, 95% confidence interval. Anti-osteoporosis drugs: Etidronate; Alendronate; RisedronateI; Ibandronate; Zoledronic acid; Alendronate; Risedronate; Denosumab. Estrogenic drugs: Conjugated estrogens Estrone; Ethinyl estradiol; Estradiol; Esterified estrogens; Estropipate estriol. Data with *P* values below 0.05 are presented in bold type.

Supplementary Table S5. ORs and 95% CIs for bone loss, additionally adjusting for menopausal status

	Osteopenia vs Normal OR (95% CI)	Osteoporosis vs Normal OR (95% CI)
hPDI		
Q1	1 (Reference)	1 (Reference)
Q2	**1.27 (1.06,1.51)**	0.90 (0.57,1.42)
Q3	**1.24 (1.02,1.50)**	0.97 (0.66,1.42)
Q4	1.22 (1.00,1.49)	0.98 (0.68,1.42)
Q5	**1.48 (1.22,1.80)**	1.05 (0.71,1.57)
Per 10-unit increment	**1.16 (1.08,1.26)**	1.02 (0.86,1.21)
PDI		
Q1	1 (Reference)	1 (Reference)

Q2	0.93 (0.77,1.13)	0.81 (0.53,1.24)
Q3	1.00 (0.82,1.22)	1.03 (0.69,1.55)
Q4	1.13 (0.92,1.37)	1.09 (0.70,1.70)
Q5	**1.20 (1.00,1.43)**	1.00 (0.66,1.51)
Per 10-unit increment	1.05 (0.95,1.15)	0.96 (0.78,1.18)
uPDI		
Q1	1 (Reference)	1 (Reference)
Q2	1.03 (0.88,1.20)	1.00 (0.68,1.47)
Q3	1.14 (0.95,1.35)	1.24 (0.84,1.83)
Q4	1.08 (0.93,1.25)	**1.44 (1.01,2.06)**
Q5	0.98 (0.79,1.22)	**1.51 (1.05,2.17)**
Per 10-unit increment	1.01 (0.93,1.10)	**1.32 (1.01,1.59)**

Note: Fully adjusted model: age, sex, and ethnicity, education, marital status, PIR, BMI, smoking status, physical exercise, hypertension, T2DM, CKD, cancer, and history of fracture. This multivariable model was additionally adjusted for menopausal status in sex. Abbreviations: OR, odds ratio; 95% CI, 95% confidence interval; PDI, plant-based diet index; hPDI, healthful plant-based diet index; uPDI, unhealthful plant-based diet index. Data with *P* values below 0.05 are presented in bold type.

Supplementary Table S6. ORs and 95% CIs for bone loss, additionally adjusting for corticosteroid usage

	**Osteopenia vs Normal**	**Osteoporosis vs Normal**
	**OR (95% CI)**	**OR (95% CI)**
hPDI		
Q1	1 (Reference)	1 (Reference)
Q2	**1.25 (1.04,1.49)**	0.89 (0.56,1.41)
Q3	**1.21 (1.00,1.47)**	0.96 (0.65,1.40)
Q4	**1.23 (1.02,1.50)**	1.02 (0.71,1.46)
Q5	**1.49 (1.23,1.80)**	1.09 (0.74,1.61)
Per 10-unit increment	**1.17 (1.08,1.26)**	1.04 (0.88,1.22)
PDI		
Q1	1 (Reference)	1 (Reference)
Q2	0.93 (0.77,1.13)	0.80 (0.52,1.22)
Q3	1.00 (0.82,1.21)	1.00 (0.67,1.49)
Q4	1.14 (0.94,1.39)	1.09 (0.71,1.68)
Q5	**1.21 (1.02,1.44)**	1.10 (0.68,1.51)
Per 10-unit increment	1.05 (0.96,1.16)	0.97 (0.79,1.19)
uPDI		
Q1	1 (Reference)	1 (Reference)
Q2	1.05 (0.91,1.22)	1.03 (0.71,1.50)
Q3	1.15 (0.97,1.37)	1.28 (0.87,1.87)
Q4	1.05 (0.91,1.22)	1.41 (0.99,2.00)
Q5	0.97 (0.79,1.20)	**1.49 (1.05,2.12)**

Per 10-unit increment	1.00 (0.92,1.08)	**1.29 (1.08,1.55)**

Note: Fully adjusted model: age, sex, and ethnicity, education, marital status, PIR, BMI, smoking status, physical exercise, hypertension, T2DM, CKD, cancer, and history of fracture. This multivariable model was additionally adjusted for corticosteroid usage. Abbreviations: OR, odds ratio; 95% CI, 95% confidence interval; PDI, plant-based diet index; hPDI, healthful plant-based diet index; uPDI, unhealthful plant-based diet index. Data with *P* values below 0.05 are presented in bold type.

Supplementary Table S7. ORs and 95% CIs for bone loss, additionally adjusting for dietary supplements

	**Osteopenia vs Normal OR (95% CI)**	**Osteoporosis vs Normal OR (95% CI)**
hPDI		
Q1	1 (Reference)	1 (Reference)
Q2	**1.24 (1.04,1.48)**	0.89 (0.56,1.41)
Q3	**1.22 (1.01,1.47)**	0.95 (0.64,1.39)
Q4	**1.25 (1.03,1.52)**	1.01 (0.70,1.45)
Q5	**1.50 (1.24,1.82)**	1.07 (0.72,1.59)
Per 10-unit increment	**1.17 (1.08,1.27)**	1.03 (0.88,1.21)
PDI		
Q1	1 (Reference)	1 (Reference)
Q2	0.93 (0.77,1.13)	0.80 (0.53,1.23)
Q3	1.00 (0.82,1.21)	1.01 (0.67,1.50)
Q4	1.14 (0.93,1.38)	1.10 (0.71,1.71)
Q5	**1.22 (1.03,1.45)**	1.02 (0.68,1.53)
Per 10-unit increment	1.06 (0.96,1.16)	0.97 (0.79,1.20)
uPDI		
Q1	1 (Reference)	1 (Reference)
Q2	1.04 (0.90,1.21)	1.03 (0.71,1.49)
Q3	1.15 (0.97,1.36)	1.28 (0.87,1.87)
Q4	1.05 (0.90,1.21)	1.39 (0.98,1.98)
Q5	0.97 (0.78,1.19)	**1.49 (1.05,2.12)**
Per 10-unit increment	0.99 (0.92,1.08)	**1.29 (1.08,1.54)**

Note: Fully adjusted model: age, sex, and ethnicity, education, marital status, PIR, BMI, smoking status, physical exercise, hypertension, T2DM, CKD, cancer, and history of fracture. This multivariable model was additionally adjusted for dietary supplements (vitamin D and calcium). Abbreviations: OR, odds ratio; 95% CI, 95% confidence interval; PDI, plant-based diet index; hPDI, healthful plant-based diet index; uPDI, unhealthful plant-based diet index. Data with *P* values below 0.05 are presented in bold type.

Supplementary Table S8. E-values for the effect of plant-based dietary on bone loss (and its lower limit of 95% CI) in fully adjusted model

E-Value for OR Estimate	E-Value for Lower limit of 95%CI	Variable	Level	Group	OR (95% CI)
1.47	1.16	hPDI	Q2 vs Q1	Osteopenia vs Normal	1.24 (1.04,1.48)
1.43	1.00	hPDI	Q3 vs Q1	Osteopenia vs Normal	1.21 (1.00,1.47)
1.47	1.11	hPDI	Q4 vs Q1	Osteopenia vs Normal	1.24 (1.02,1.51)

1.75	1.47	hPDI	Q5 vs Q1	Osteopenia vs Normal	1.50 (1.24,1.81)
1.38	1.24	hPDI	Per 10-unit increment	Osteopenia vs Normal	1.17 (1.08,1.27)
1.44	1.14	PDI	Q5 vs Q1	Osteopenia vs Normal	1.22 (1.03,1.45)
2.32	1.24	uPDI	Q5 vs Q1	Osteoporosis vs Normal	1.48 (1.04,2.11)
1.90	1.37	uPDI	Per 10-unit increment	Osteoporosis vs Normal	1.29 (1.08,1.54)

Note: Fully adjusted model: age, sex, and ethnicity, education, marital status, PIR, BMI, smoking status, physical exercise, hypertension, T2DM, CKD, cancer, and history of fracture. Abbreviations: OR, odds ratio; 95% CI, 95% confidence interval; PDI, plant-based diet index; hPDI, healthful plant-based diet index; uPDI, unhealthful plant-based diet index.

	PDI	hPDI	uPDI
Plant-based Food Groups			
Healthy			
Whole grains	Postive scores	Postive scores	Reverse scores
Fruits	Postive scores	Postive scores	Reverse scores
Vegetables	Postive scores	Postive scores	Reverse scores
Nuts	Postive scores	Postive scores	Reverse scores
Legumes	Postive scores	Postive scores	Reverse scores
Tea and coffee	Postive scores	Postive scores	Reverse scores
Less healthy			

Fruit juices	Postive scores	Reverse scores	Postive scores
Refined grain	Postive scores	Reverse scores	Postive scores
Potatoes	Postive scores	Reverse scores	Postive scores
Sugar	Postive scores	Reverse scores	Postive scores
Animal-based Food Groups			
Animal fat	Reverse scores	Reverse scores	Reverse scores
Dairy	Reverse scores	Reverse scores	Reverse scores
Egg	Reverse scores	Reverse scores	Reverse scores
Seafood	Reverse scores	Reverse scores	Reverse scores
Meat	Reverse scores	Reverse scores	Reverse scores
